# Supplementary material for: Exploring beyond clinical routine SARS-CoV-2 serology using MultiCoV-Ab to evaluate endemic coronavirus cross-reactivity
Source: Nat Commun. 2021 Feb 19;12:1152. doi: 10.1038/s41467-021-20973-3 (PMC7896075; doi:10.1038/s41467-021-20973-3)
Supplement: Supplementary file 1 — Supplementary Information [file 41467_2021_20973_MOESM1_ESM.pdf]

Supplementary Information

Exploring beyond clinical routine SARS-CoV-2 serology using MultiCoV-Ab to evaluate endemic coronavirus cross-reactivity

Matthias Becker[1,#], Monika Strengert[2,3,#], Daniel Junker[1], Philipp D. Kaiser[1], Tobias Kerrinnes[4], Bjoern Traenkle[1,5], Heiko Dinter[1,5], Julia Häring[1], Stéphane Ghozzi[2], Anne Zeck[1], Frank Weise[1], Andreas Peter[6,7,8], Sebastian Hörber[6,7,8], Simon Fink[1], Felix Ruoff[1], Alex Dulovic[1], Tamam Bakchoul[9], Armin Baillot[10], Stefan Lohse[11], Markus Cornberg[12], Thomas Illig[13], Jens Gottlieb[14,15], Sigrun Smola[11], André Karch[16], Klaus Berger[16], Hans-Georg Rammensee[17,18,19], Katja Schenke-Layland[1,19,20,21], Annika Nelde[17,19,22], Melanie Märklin[19,21], Jonas S. Heitmann[19,21], Juliane S. Walz[17,19,23,22], Markus Templin[1], Thomas O. Joos[1], Ulrich Rothbauer[1,5,#], Gérard Krause[2,3], Nicole Schneiderhan-Marra[1,*]

Affiliations

[1] NMI Natural and Medical Sciences Institute at the University of Tübingen, Reutlingen, Germany

[2] Department of Epidemiology, Helmholtz Centre for Infection Research, Braunschweig, Germany

[3] TWINCORE GmbH, Centre for Experimental and Clinical Infection Research, a joint venture of the Hannover Medical School and the Helmholtz Centre for Infection Research, Hannover, Germany

[4] Helmholtz-Institute for RNA-based Infection Research (HIRI), Würzburg, Germany

[5] Pharmaceutical Biotechnology, University of Tübingen, Germany

[6] Institute for Clinical Chemistry and Pathobiochemistry, Department for Diagnostic Laboratory Medicine, University Hospital Tübingen, Tübingen, Germany

[7] Institute for Diabetes Research and Metabolic Diseases of the Helmholtz Center Munich at the University of Tübingen, Tübingen, Germany

[8] German Center for Diabetes Research (DZD), München-Neuherberg, Germany

[9] Institute for Clinical and Experimental Transfusion Medicine, University Hospital Tübingen, Tübingen, Germany

[10] Niedersächsisches Landesgesundheitsamt, Department of Virology/Serology, Hannover, Germany

[11] Institute of Virology, Saarland University Medical Center, Homburg/Saar, Germany

[12] Department of Gastroenterology, Hepatology, Endocrinology, Hannover Medical School, Hannover, Germany; Centre for Individualized Infection Medicine (CiiM), Hannover, Germany

[13] Hannover Unified Biobank (HUB), Hannover Medical School, Hannover, Germany

[14] Department of Respiratory Medicine, Hannover Medical School, Hannover Germany

[15] Biomedical Research in End-stage and obstructive Lung Disease Hannover (BREATH), member of the German Center for Lung Research (DZL), Hannover, Germany

[16] Institute of Epidemiology and Social Medicine, University of Münster, Münster, Germany

[17] Institute for Cell Biology, Department of Immunology, University of Tübingen, Tübingen, Germany

[18] German Cancer Consortium (DKTK) and German Cancer Research Center (DKFZ), partner site Tübingen, Tübingen, Germany.

[19] Cluster of Excellence iFIT (EXC2180) "Image-Guided and Functionally Instructed Tumor Therapies", University of Tübingen, Tübingen, Germany

[20] Department of Women's Health, Research Institute for Women's Health, Eberhard-Karls-University, Tübingen, Germany

[21] Department of Medicine/Cardiology, Cardiovascular Research Laboratories, David Geffen School of Medicine at UCLA, Los Angeles, CA, USA

[22] Clinical Collaboration Unit Translational Immunology, German Cancer Consortium (DKTK), Department of Internal Medicine, University Hospital Tübingen, Tübingen, Germany

[23] Dr. Margarete Fischer-Bosch Institute of Clinical Pharmacology (IKP) and Robert Bosch Center for Tumor Diseases (RBCT), both Stuttgart, Germany

[#] These authors contributed equally to this work.

* Corresponding author:

Dr. Nicole Schneiderhan-Marra

Markwiesenstrasse 55, 72770 Reutlingen, Germany

Phone: 0049 7121 51530 815

Fax: 0049 7121 51530 16

E-Mail: Nicole.Schneiderhan@nmi.de

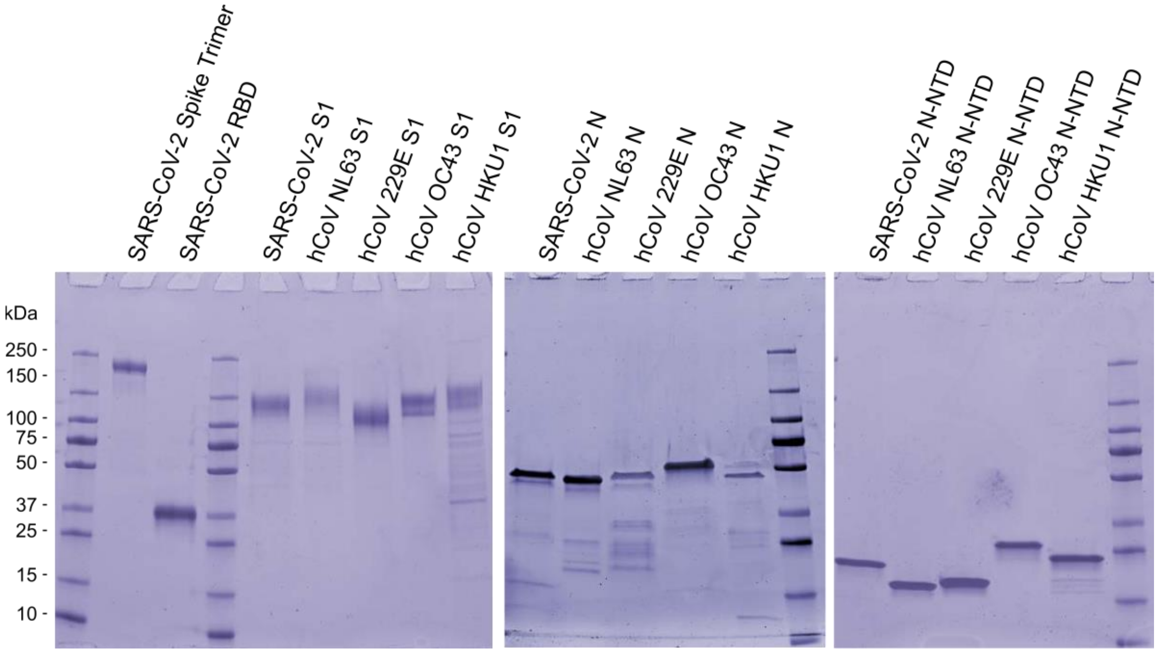

Supplementary Figure 1

SDS-PAGE analysis of the recombinant viral antigens used in this study. To test for purity and integrity 1 - 2 µg of indicated recombinant proteins were boiled in reducing SDS-sample buffer and subjected to a gradient (4 – 20 %) SDS-PAGE followed by Coomassie staining. SARS-CoV-2 Spike Trimer, SARS-CoV-2 RBD and the S1-domains of SARS-CoV-2, hCoV-NL63, hCoV-229E, hCoV-OC43 and hCoV-HKU1 were produced in ExpiHEK™ cells. Nucleocapsid (N) and N-terminal domain of nucleocapsid (N-NTD) of SARS-CoV-2, hCoV-NL63, hCoV-229E, hCoV-OC43 and hCoV-HKU1 were produced in *E.coli*. The image is an assembly of all expressed proteins and is representative for single protein purity after purification. Source data are provided as a Source Data file.

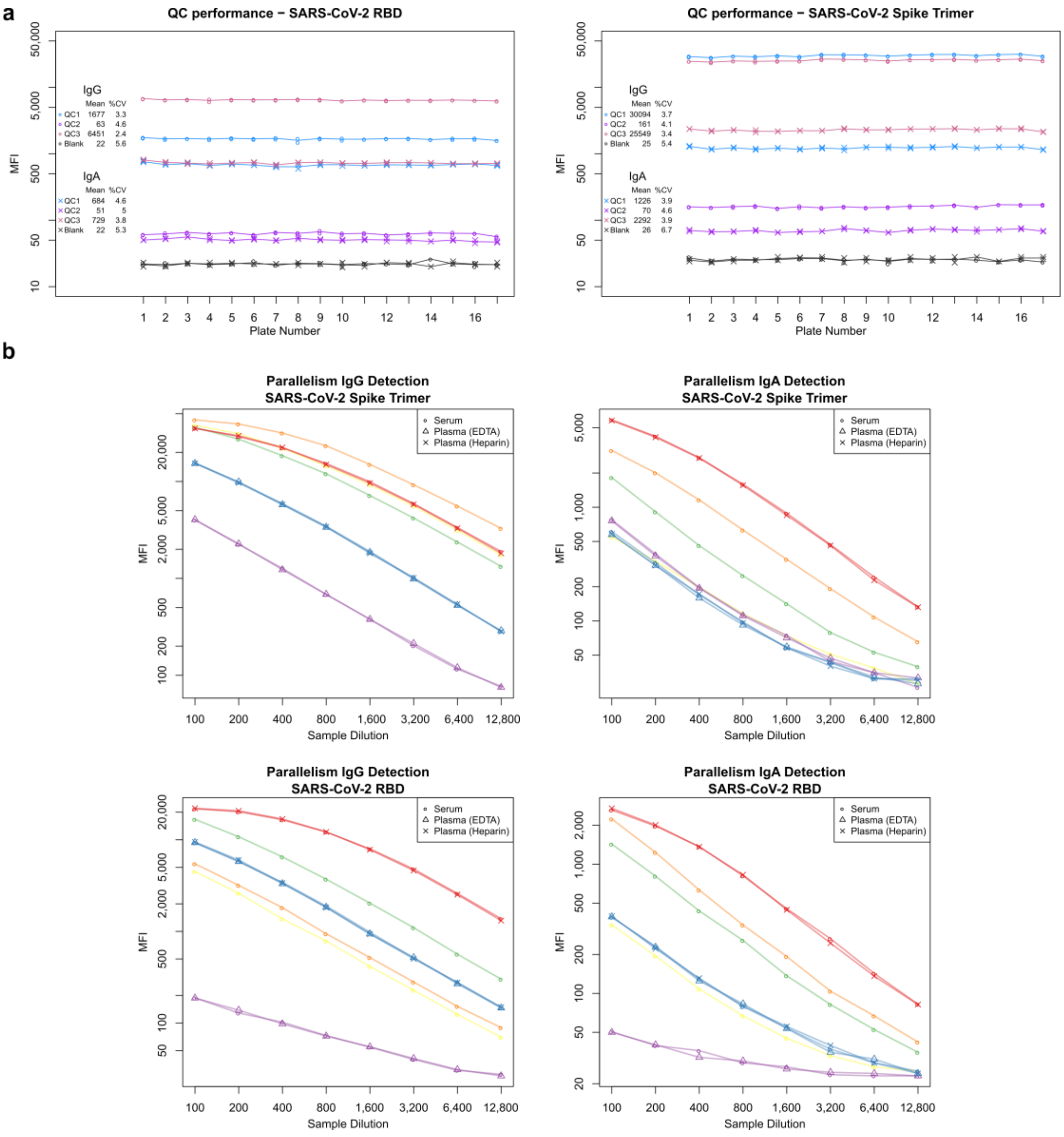

Supplementary Figure 2

a, Three quality control (QC) samples, as well as a sample of assay buffer (blank sample) were processed in duplicates on every plate. Performance across 17 assay runs is depicted and mean and %CVs are shown on the left side (n=34). For plate 14, a processing error lead to exclusion of one blank sample from this evaluation. **b,** To assess parallelism of signals from different samples, 6 unique serum samples were processed over a dilution series of 8 steps from 1:100 to 1:12,800. For 3 samples, paired plasma (EDTA and/or Heparin) were available and processed together. For IgG and IgA detection of Spike Trimer and RBD, MFI are plotted against sample dilution. Color indicates unique sample and shapes indicate sample type. The data represent a single measurement per sample and dilution (n=1). Source data are provided as a Source Data file.

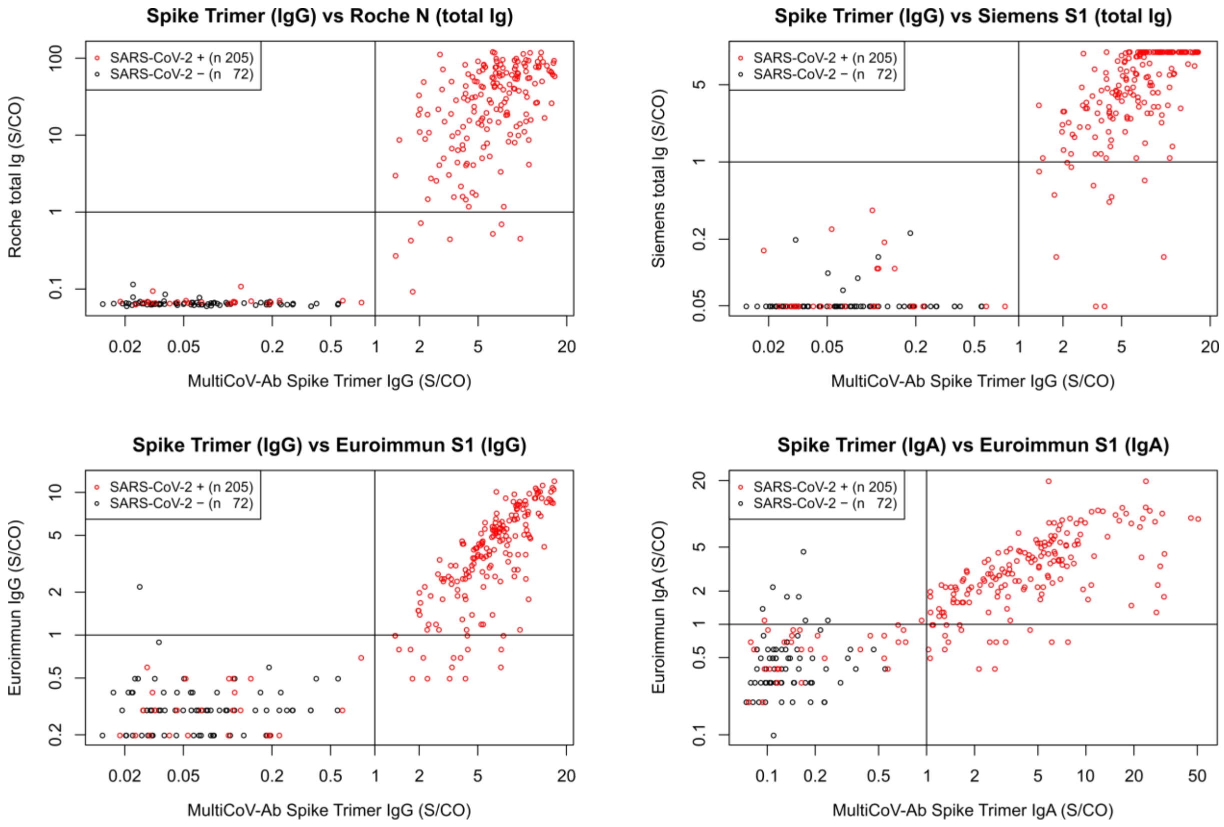

Supplementary Figure 3

Scatterplots of sample set with defined SARS-CoV-2 infection status (infected: red, n=205; uninfected: black, n=72) to compare performance of the MultiCoV-Ab Spike Trimer vs indicated antigens of commercial SARS-CoV-2 test kits. Signals are depicted as Signal to cut-off ratios (S/CO) on a logarithmic scale. Lines indicate the respective cut-off values as defined by the manufacturer to determine positive and negative test results. Source data are provided as a Source Data file.

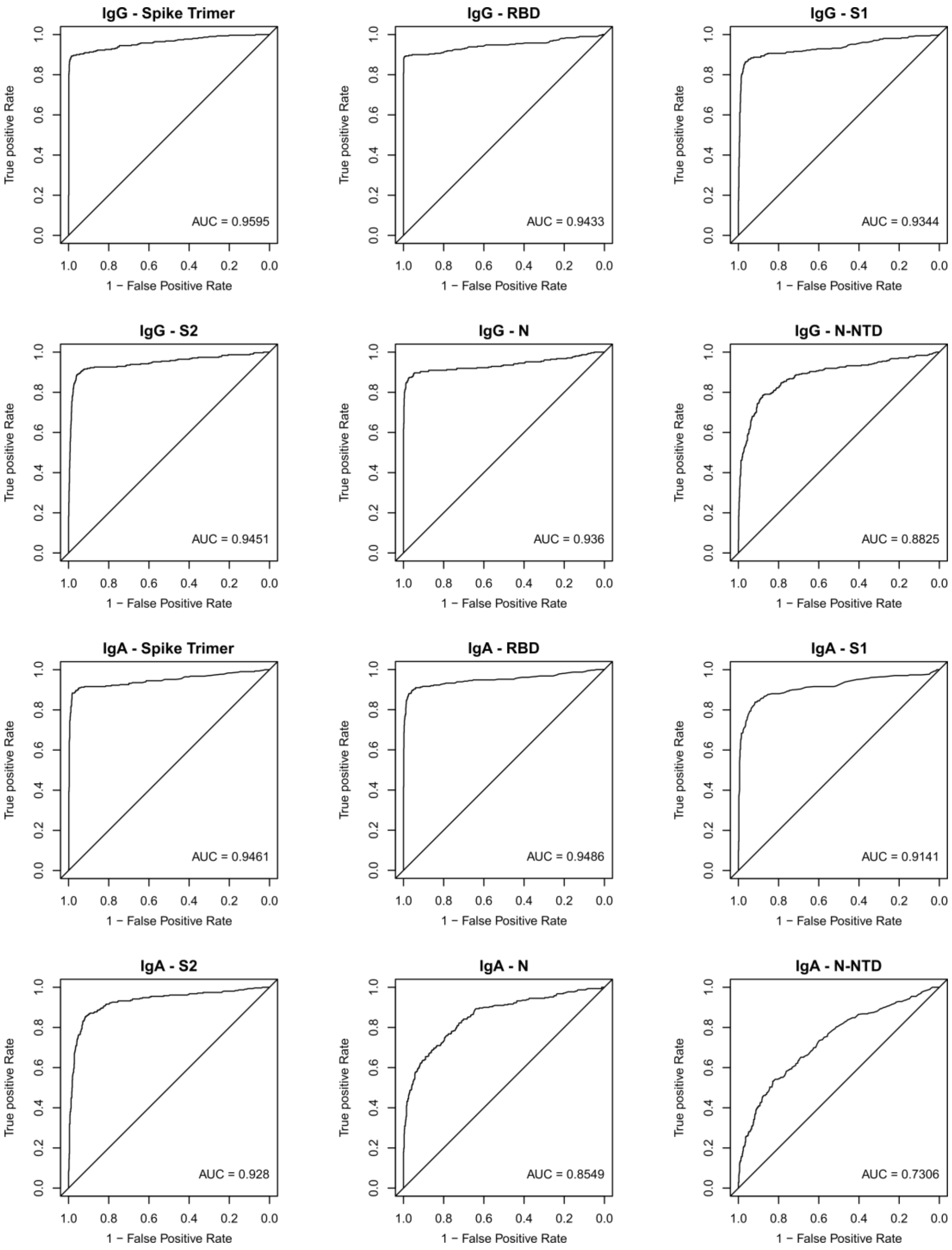

Supplementary Figure 4

ROC analysis[1,2] for IgG and IgA detection of SARS-CoV-2 antigens based on the extended sample set of 866 uninfected and 310 infected samples used for clinical validation for MultiCoV-Ab. True positive rate is displayed against 1 - false positive rate, corresponding to sensitivity and specificity at a given cut-off. AUC-values indicating individual antigen performance are shown. Source data are provided as a Source Data file.

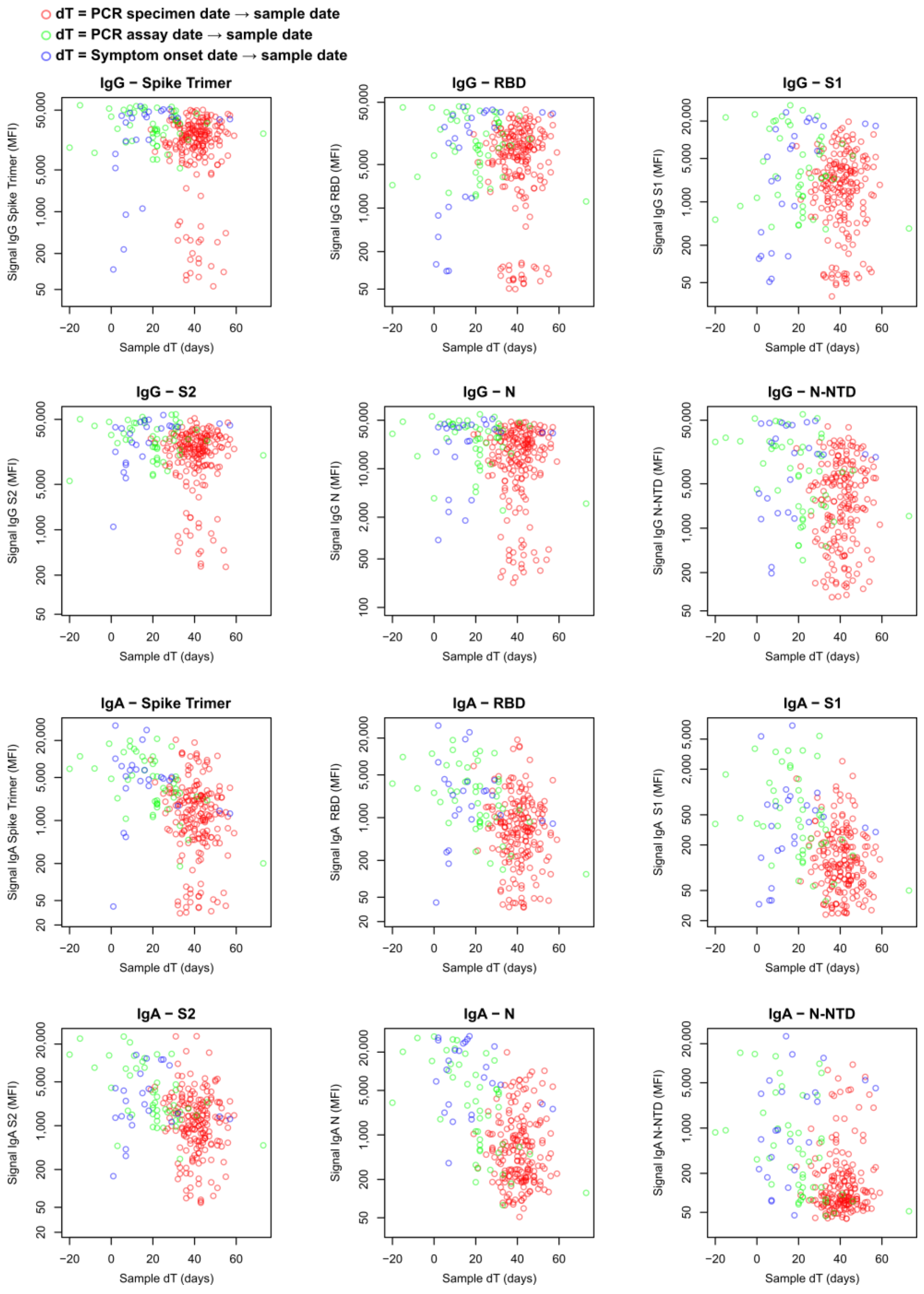

Supplementary Figure 5

Impact of sample time on antibody response is visualized by scatter plots. Sample dT in days is displayed against the observed MFI signal per antigen. Definition of dT is not consistent as samples measured in this study were taken from various sources. dT was calculated from the day of a positive PCR result (red circles), from the day of the PCR test itself (green circles) or from the day of symptom onset (blue circles). Source data are provided as a Source Data file.

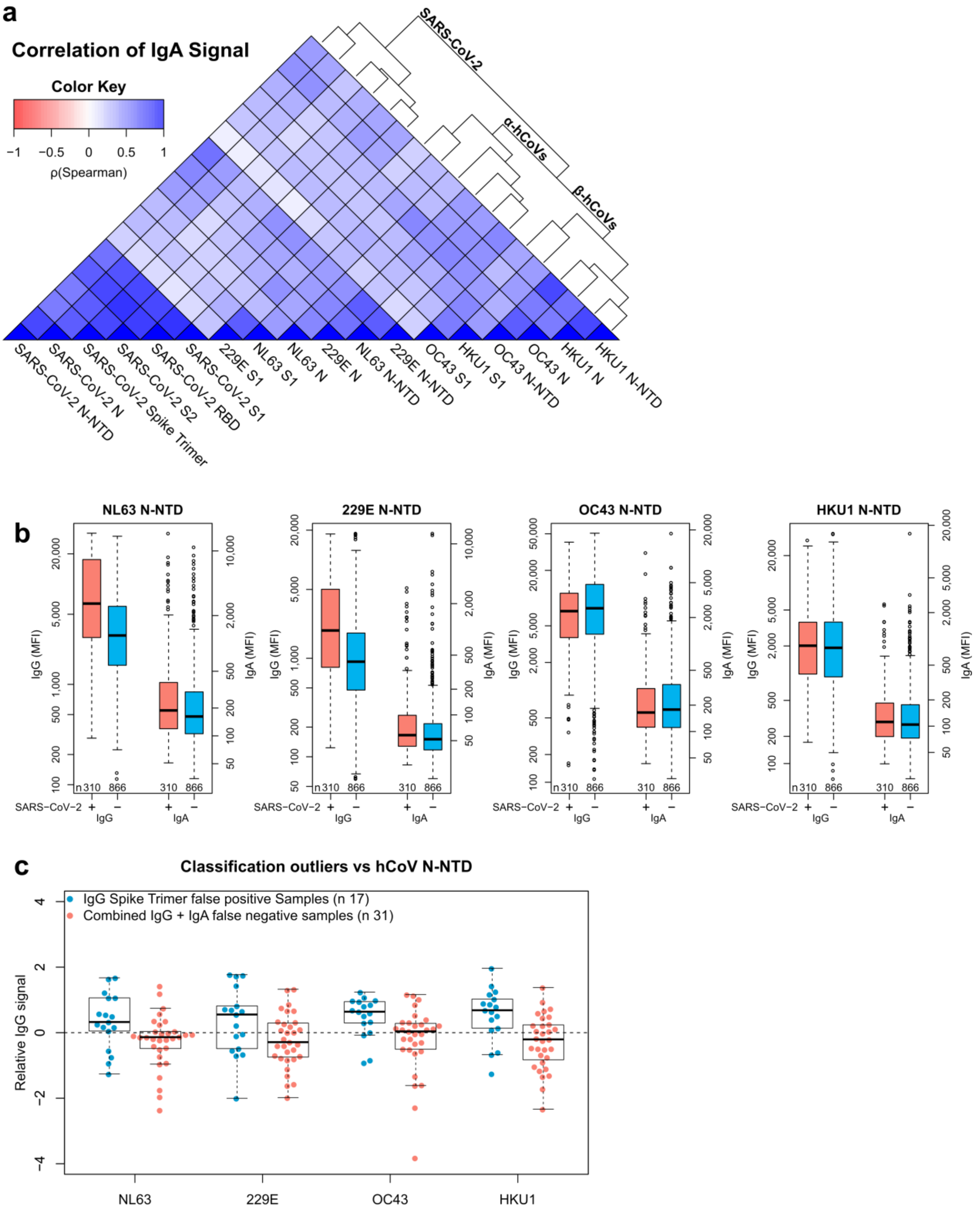
a
Correlation of IgA Signal
Color Key
−1 −0.5 0 0.5 1
ρ(Spearman)
SARS-CoV-2
α-hCoVs
β-hCoVs
SARS-CoV-2 N-NTD
SARS-CoV-2 N
SARS-CoV-2 Spike Trimer
SARS-CoV-2 S2
SARS-CoV-2 RBD
SARS-CoV-2 S1
229E S1
NL63 S1
NL63 N
229E N
NL63 N-NTD
229E N-NTD
OC43 S1
HKU1 S1
OC43 N-NTD
OC43 N
HKU1 N
HKU1 N-NTD
b
NL63 N-NTD
229E N-NTD
OC43 N-NTD
HKU1 N-NTD
IgG (MFI)
IgA (MFI)
n 310 866 310 866
SARS−CoV−2 + − + −
IgG
IgA
c
Classification outliers vs hCoV N-NTD
IgG Spike Trimer false positive Samples (n 17)
Combined IgG + IgA false negative samples (n 31)
Relative IgG signal
NL63
229E
OC43
HKU1

Supplementary Figure 6

a, Correlation of IgA response for the entire sample set (n=1176) is visualized as heatmap based on Spearman's ρ coefficient; dendrogram on the right side displays antigens after hierarchical clustering was performed. **b,** Immune response (IgG and IgA) towards hCoV N-NTD proteins are presented as Box-Whisker plots of sample MFI on a logarithmic scale for SARS-CoV-2-infected (red, n=310) and uninfected (blue, n=866) individuals. Box represents the median and the 25th and 75th percentiles, whiskers show the largest and smallest values. Outliers determined by 1.5 times IQR of log-transformed data are depicted as circles. **c,** Relative levels of IgG-specific immune response towards hCoV N-NTD proteins are presented as Box-Whisker plots / stripchart overlays of log-transformed and per-antigen scaled and centred MFI for the sample subsets of Spike Trimer false positives (blue, n=17) and combined IgG + IgA false negatives (red, n=31). Box represents the median and the 25th and 75th percentiles, whiskers show the largest and smallest values, excluding outliers as determined by 1.5 times IQR. Source data are provided as a Source Data file.

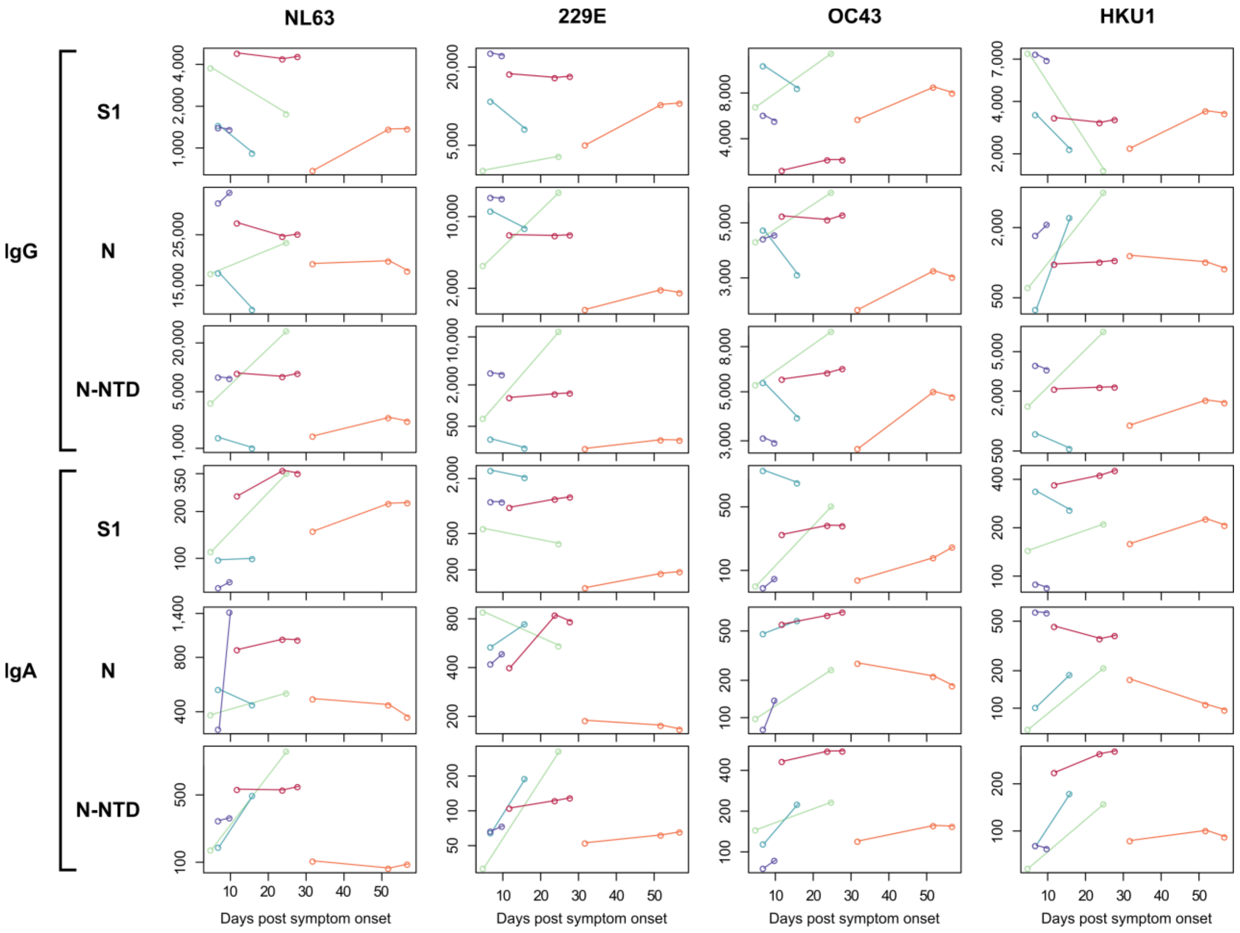

Supplementary Figure 7

Kinetic of hCoV antigen-specific IgA and IgG responses is shown for indicated days after symptom onset for the three used hCoV antigens across five different patients. Colored lines indicated kinetic of respective SARS-CoV-2 antigen per patient. Source data are provided as a Source Data file.

Supplementary Table 1 | Assay Variance and LOD

Intra- and inter-assay variance were determined by repeated measurement of QC samples and blank sample as replicates on one plate and in duplicates over 17 plates, respectively. Standard deviation relative to mean (%CV) is given for each antigen. A limit of detection (LOD) was calculated from 24 blank sample replicates on the same plate as the mean MFI + 3 times standard deviation.

			SARS-CoV-2						hCoV NL63			hCoV 229E			hCoV OC43			hCoV HKU1		
			Spike Trimer	RBD	S1	S2	N	N-NTD	S1	N	N-NTD	S1	N	N-NTD	S1	N	N-NTD	S1	N	N-NTD
Inter-assay variance (%CV) n = 34, duplicates, 17 plates	IgG	QC1	3.7	3.3	3.4	3.7	2.8	7.4	3.5	3.2	4.4	3.3	2.8	5.2	3.1	6.0	4.4	3.4	4.7	5.4
		QC2	4.1	4.6	6.9	3.4	5.3	4.8	3.0	2.2	6.3	2.4	2.1	6.7	2.7	4.5	2.3	2.7	5.1	2.8
		QC3	3.4	2.4	2.3	3.6	2.1	4.6	3.1	2.5	3.5	2.9	2.0	4.7	2.9	6.4	4.6	3.2	3.2	3.5
		Blank	5.4	5.6	6.7	6.4	5.6	6.1	6.3	7.1	5.7	9.1	6.1	6.1	5.6	7.3	4.1	4.9	6.1	8.3
	IgA	QC1	3.9	4.6	4.9	4.0	5.1	5.0	4.2	3.6	3.9	4.0	5.3	7.4	4.3	7.4	5.0	4.2	6.0	5.0
		QC2	4.6	5.0	5.1	3.9	3.9	4.2	3.7	2.4	7.6	2.9	2.2	6.0	4.1	16.4	4.2	4.3	5.5	3.9
		QC3	3.9	3.8	4.5	3.4	3.4	4.8	3.9	2.8	4.0	3.0	5.1	4.5	3.6	6.1	4.1	3.7	4.5	5.1
		Blank	6.7	5.3	8.2	6.3	5.3	5.3	3.3	5.0	5.0	6.7	7.0	6.1	5.3	7.1	4.7	6.0	6.8	6.3
Intra-assay variance (%CV) n = 24	IgG	QC1	2.5	1.9	2.0	2.1	1.8	2.1	2.4	1.7	2.8	2.0	2.7	3.2	1.9	2.0	2.2	2.7	2.4	2.2
		QC2	5.9	4.3	4.1	2.8	2.7	3.2	1.9	1.9	2.6	2.0	2.2	2.7	2.2	1.6	2.1	2.5	3.1	2.5
		QC3	1.6	4.3	5.1	1.9	1.9	4.5	4.2	1.7	3.2	3.3	4.1	5.7	3.2	3.1	5.5	5.6	6.0	8.4
		Blank	6.0	5.6	5.2	5.8	5.2	4.2	5.0	4.8	4.8	7.3	6.2	6.3	6.5	6.2	4.4	6.1	6.0	6.2
	IgA	QC1	2.5	3.3	5.2	3.8	3.7	4.2	3.2	2.3	2.2	2.0	4.8	4.7	2.9	4.7	3.4	3.3	4.5	4.3
		QC2	4.8	5.7	5.7	3.2	4.1	4.3	3.4	2.0	5.7	2.1	2.1	6.1	3.0	3.1	1.9	3.9	6.4	3.8
		QC3	3.1	4.7	5.5	3.0	4.1	4.4	3.7	2.7	3.7	2.7	5.4	5.8	2.6	4.1	3.1	2.4	4.5	4.3
		Blank	5.8	5.3	6.3	5.0	5.4	5.5	4.5	5.6	5.3	7.2	6.3	7.0	6.7	9.5	7.3	5.2	8.8	7.0
LOD (MFI) n = 24	IgG		32	26	23	29	38	33	29	28	26	65	35	24	33	30	33	37	33	25
	IgA		31	26	26	27	37	32	57	28	40	28	36	22	35	28	32	33	39	28

Supplementary Table 2 | Complete overview of study sample set divided into columns by age groups and sex.
Samples from SARS-CoV-2-infected donors are further split up by hospitalization status. Age and gender of patients from which multiple samples were available for time course analyses are indicated. SARS-CoV-2-uninfected samples are further divided into samples drawn during the pandemic, which was defined as all samples taken on 01.01.2020 or later, and pre-pandemic samples. 147 samples with previous hCoV infection were included in the SARS-CoV-2-uninfected group. Detailed diagnosis of hCoV subspecies is indicated where available. Other sample conditions for special groups of uninfected samples are listed. NA: Information was not available.

Age	≤39		40-59		≥60		NA			∑
n	299 (25.4 %)		241 (20.5 %)		475 (40.4 %)		161 (13.7 %)			1176
Sex	male	female	male	female	male	female	male	female	NA	
n	139 (11.8 %)	160 (13.6 %)	144 (12.2 %)	97 (8.2 %)	271 (23.0 %)	204 (17.3 %)	5 (0.4 %)	3 (0.3 %)	153 (13.0 %)	1176
SARS-CoV-2-infected (total)	60 (19.4 %)	51 (16.5 %)	71 (22.9 %)	63 (20.3 %)	42 (13.5 %)	17 (5.5 %)	3 (1.0 %)	3 (1.0 %)	0 (0.0 %)	310
Hospitalized (for COVID19)	6 (10.9 %)	2 (3.6 %)	14 (25.5 %)	6 (10.9 %)	23 (41.8 %)	4 (7.3 %)	0 (0.0 %)	0 (0.0 %)	0 (0.0 %)	55
Non-Hospitalized	52 (25.0 %)	43 (20.7 %)	49 (23.6 %)	43 (20.7 %)	13 (6.3 %)	8 (3.8 %)	0 (0.0 %)	0 (0.0 %)	0 (0.0 %)	208
Hospitalisation NA	2 (4.3 %)	6 (12.8 %)	8 (17.0 %)	14 (29.8 %)	6 (12.8 %)	5 (10.6 %)	3 (6.4 %)	3 (6.4 %)	0 (0.0 %)	47
Patients with time series	2 (40.0 %)	0 (0.0 %)	0 (0.0 %)	0 (0.0 %)	2 (40.0 %)	1 (20.0 %)	0 (0.0 %)	0 (0.0 %)	0 (0.0 %)	5
SARS-CoV-2-uninfected (total)	79 (9.1 %)	109 (12.6 %)	73 (8.4 %)	34 (3.9 %)	229 (26.4 %)	187 (21.6 %)	2 (0.2 %)	0 (0.0 %)	153 (17.7 %)	866
Sample during pandemic	10 (15.4 %)	10 (15.4 %)	12 (18.5 %)	14 (21.5 %)	7 (10.8 %)	5 (7.7 %)	1 (1.5 %)	0 (0.0 %)	6 (9.2 %)	65
Sample pre-pandemic	69 (8.6 %)	99 (12.4 %)	61 (7.6 %)	20 (2.5 %)	222 (27.7 %)	182 (22.7 %)	1 (0.1 %)	0 (0.0 %)	147 (18.4 %)	801
Previous hCoV Infection	19 (12.9 %)	18 (12.2 %)	45 (30.6 %)	20 (13.6 %)	29 (19.7 %)	16 (10.9 %)	0 (0.0 %)	0 (0.0 %)	0 (0.0 %)	147
confirmed NL63	2 (20.0 %)	0 (0.0 %)	3 (30.0 %)	1 (10.0 %)	2 (20.0 %)	2 (20.0 %)	0 (0.0 %)	0 (0.0 %)	0 (0.0 %)	10
confirmed 229	5 (25.0 %)	1 (5.0 %)	4 (20.0 %)	1 (5.0 %)	5 (25.0 %)	4 (20.0 %)	0 (0.0 %)	0 (0.0 %)	0 (0.0 %)	20
confirmed OC43	0 (0.0 %)	1 (3.7 %)	14 (51.9 %)	1 (3.7 %)	6 (22.2 %)	5 (18.5 %)	0 (0.0 %)	0 (0.0 %)	0 (0.0 %)	27
confirmed HKU1	3 (20 %)	1 (6.7 %)	4 (26.7 %)	2 (13.3 %)	5 (33.3 %)	0 (0.0 %)	0 (0.0 %)	0 (0.0 %)	0 (0.0 %)	15
unknown hCoV	9 (12 %)	15 (20.0 %)	20 (26.7 %)	15 (20.0 %)	11 (14.7 %)	5 (6.7 %)	0 (0.0 %)	0 (0.0 %)	0 (0.0 %)	75
Pregnant	0 (0.0 %)	9 (90.0 %)	0 (0.0 %)	1 (10.0 %)	0 (0.0 %)	0 (0.0 %)	0 (0.0 %)	0 (0.0 %)	0 (0.0 %)	10
RF/HAMA samples	0 (0.0 %)	0 (0.0 %)	0 (0.0 %)	0 (0.0 %)	0 (0.0 %)	0 (0.0 %)	0 (0.0 %)	0 (0.0 %)	6 (100 %)	6
PCT > 3 ng/mL	0 (0.0 %)	0 (0.0 %)	0 (0.0 %)	0 (0.0 %)	0 (0.0 %)	0 (0.0 %)	0 (0.0 %)	0 (0.0 %)	21 (100 %)	21
Neuroinflammatory disease	6 (40.0 %)	6 (40.0 %)	1 (6.7 %)	0 (0.0 %)	1 (6.7 %)	1 (6.7 %)	0 (0.0 %)	0 (0.0 %)	0 (0.0 %)	15

Supplementary Table 3 - Percentage Identity of sequence alignments of used hCoV and corresponding SARS-CoV-2. Alignments were calculated using version 1.2.4. of Clustal Omega[3]. Sequences of constructs used in alignment are provided as a Source Data file.

		% of sequence identity of corresponding antigen				
Protein	Identifier	hCoV-NL63	hCoV-229E	SARS-CoV-2	hCoV-OC43	hCoV-HKU1
hCoV-NL63 S1	APF29071.1	100.0	50.2	17.8	19.7	18.1
hCoV-229E S1	APT69883.1	50.2	100.0	18.5	20.2	18.7
SARS-CoV-2 S1	QHD43416.1	17.8	18.5	100.0	24.2	24.2
hCoV-OC43 S1	AVR40344.1	19.7	20.2	24.2	100.0	58.0
hCoV-HKU1 S1	AGW27881.1	18.1	18.7	24.2	58.0	100.0
hCoV-NL63 N	YP_003771.1	100.0	47.5	30.9	29.0	29.7
hCoV-229E N	NP_073556.1	47.5	100.0	30.4	30.1	32.2
SARS-CoV-2 N	QHD43423.2	30.9	30.4	100.0	37.1	36.7
hCoV-OC43 N	YP_009555245.1	29.0	30.1	37.1	100.0	65.5
hCoV-HKU1 N	YP_173242.1	29.7	32.2	36.7	65.5	100.0
hCoV-NL63 N-NTD	YP_003771.1	100.0	63.4	35.3	34.4	35.8
hCoV-229E N-NTD	NP_073556.1	63.4	100.0	38.2	37.9	39.2
SARS-CoV-2 N-NTD	QHD43423.2	35.3	38.2	100.0	42.0	42.8
hCoV-OC43 N-NTD	YP_009555245.1	34.4	37.9	42.0	100.0	68.3
hCoV-HKU1 N-NTD	YP_173242.1	35.8	39.2	42.8	68.3	100.0

Supplementary Table 4 | Overview of primers used in this study with respective sequence.

Primer	Sequence
pRSET2b down-for	GGTAAGCTTGATCCGGCTGCTAA
SARS-CoV2_NTD-rev	GGGAAGCTTACTCAGCATAGAAGCCCTTTGG
OC43_NTD-rev	GGGAAGCTTATTCGATATAATAGCCCTGCGG
NL63_NTD-rev	GGGAAGCTTATTCAACAACGCTCAGTTCCG
229E_NTD-rev	GGGAAGCTTATTCAACAACGGTAACACCATTC
HKU1_NTD-rev	GGGAAGCTTATTCCACATAGTAGCCCTGAGGC
S1 CoV2-for	CTTCTGGCGTGTGACCGG
S1 CoV2-rev	GTTGCGGCCGCTTAGTGGTGGTGGTGGTGGTGGGGGCTGTTTGTCTGTGTCTG

Supplementary Table 5 | Overview of antigens used in this study.

Construct	Manufacturer	Sequence Identifier	Fragment	Mutations	Expression system	Tag	Tag position
SARS-CoV-2 Spike Trimer	In-house expressed	QHD43416.1	1-1213	^{682}RRAR to A, K986P and V987P	Expi293	Thrombin cleavage-site/ T4 foldon/ His_6	C-terminus
SARS-CoV-2 RBD	In-house expressed	QHD43416.1	1-14 + 319-541		Expi293	His_6	C-terminus
SARS-CoV-2 S2	Sino Biological #40590-V08B	YP_009724390.1	686-1213		Baculovirus-Insect cells	His_6	C-terminus
SARS-CoV-2 S1	In-house expressed	QHD43416.1	1-681		Expi293	His_6	C-terminus
hCoV-OC43 S1	In-house expressed	AVR40344.1	1-760		Expi293	His_6	C-terminus
hCoV-NL63 S1	In-house expressed	APF29071.1	1-744		Expi293	His_6	C-terminus
hCoV-229E S1	In-house expressed	APT69883.1	1-561		Expi293	His_6	C-terminus
hCoV-HKU1 S1	In-house expressed	AGW27881.1	1-755		Expi293	His_6	C-terminus
SARS-CoV-2 N	Aalto Bioreagents #CK 6406-b	NA	full length		*E. coli*	His_6	C-terminus
SARS-CoV-2 N-NTD	In-house expressed	QHD43423.2	1-174		*E.coli* BL21	His_6	N-terminus
hCoV-OC43 N	In-house expressed	YP_009555245.1	1-448		*E.coli* BL21	His_6	N-terminus
hCoV-OC43 N-NTD	In-house expressed	YP_009555245.1	1-189		*E.coli* BL21	His_6	N-terminus
hCoV-NL63 N	In-house expressed	YP_003771.1	1-377		*E.coli* BL21	His_6	N-terminus
hCoV-NL63 N-NTD	In-house expressed	YP_003771.1	1-139		*E.coli* BL21	His_6	N-terminus
hCoV-229E N	In-house expressed	NP_073556.1	1-389		*E.coli* BL21	His_6	N-terminus
hCoV-229E N-NTD	In-house expressed	NP_073556.1	1-141		*E.coli* BL21	His_6	N-terminus
hCoV-HKU1 N	In-house expressed	YP_173242.1	1-441		*E.coli* BL21	His_6	N-terminus
hCoV-HKU1 N-NTD	In-house expressed	YP_173242.1	1-188		*E.coli* BL21	His_6	N-terminus

For commercial antigens, catalogue number is given and information is provided as available from the data sheets. NA: Information was not available.

Supplementary References

1 Fawcett, T. An introduction to ROC analysis. *Pattern recognition letters* **27**, 861-874 (2006).

2 Zou, K. H., O'Malley, A. J. & Mauri, L. Receiver-operating characteristic analysis for evaluating diagnostic tests and predictive models. *Circulation* **115**, 654-657 (2007).

3 Madeira, F. *et al.* The EMBL-EBI search and sequence analysis tools APIs in 2019. *Nucleic acids research* **47**, W636-W641 (2019).
