# Supplementary material for: Exploring beyond clinical routine SARS-CoV-2 serology using MultiCoV-Ab to evaluate endemic coronavirus cross-reactivity
Source: Nat Commun. 2021 Feb 19;12:1152. doi: 10.1038/s41467-021-20973-3 (PMC7896075; doi:10.1038/s41467-021-20973-3)
Supplement: Supplementary file 3 — Reporting Summary [file 41467_2021_20973_MOESM3_ESM.pdf]

nature research

Corresponding author(s): Nicole Schneiderhan-Marra

Last updated by author(s): Dec 19, 2020

Reporting Summary

Nature Research wishes to improve the reproducibility of the work that we publish. This form provides structure for consistency and transparency in reporting. For further information on Nature Research policies, see our Editorial Policies and the Editorial Policy Checklist.

Statistics

For all statistical analyses, confirm that the following items are present in the figure legend, table legend, main text, or Methods section.

n/a	Confirmed	
☐	☒	The exact sample size (*n*) for each experimental group/condition, given as a discrete number and unit of measurement
☐	☒	A statement on whether measurements were taken from distinct samples or whether the same sample was measured repeatedly
☐	☒	The statistical test(s) used AND whether they are one- or two-sided<br>*Only common tests should be described solely by name; describe more complex techniques in the Methods section.*
☒	☐	A description of all covariates tested
☒	☐	A description of any assumptions or corrections, such as tests of normality and adjustment for multiple comparisons
☐	☒	A full description of the statistical parameters including central tendency (e.g. means) or other basic estimates (e.g. regression coefficient) AND variation (e.g. standard deviation) or associated estimates of uncertainty (e.g. confidence intervals)
☐	☒	For null hypothesis testing, the test statistic (e.g. *F*, *t*, *r*) with confidence intervals, effect sizes, degrees of freedom and *P* value noted<br>*Give P values as exact values whenever suitable.*
☒	☐	For Bayesian analysis, information on the choice of priors and Markov chain Monte Carlo settings
☒	☐	For hierarchical and complex designs, identification of the appropriate level for tests and full reporting of outcomes
☐	☒	Estimates of effect sizes (e.g. Cohen's *d*, Pearson's *r*), indicating how they were calculated

Our web collection on statistics for biologists contains articles on many of the points above.

Software and code

Policy information about availability of computer code

Data collection	Luminex FLEXMAP 3D instrument with xPONENT Software 4.3, BEP2000 Advance System, Cobas e411 analyzer, ADVIA Centaur XPT, Prometheus instrument with PR.ThermControl software v.2.0.4, Typhoon Trio instrument with Thyphoon Scanner Control software v5.0. Meta-data for pre-exitsting samples were provided with the samples.
Data analysis	Data analysis and visualization was performed with R Studio (Version 1.2.5001, using R version 3.6.1). Analysis code has been deposited on GitHub: https://github.com/BeckerMatthias/MULTICOV-AB_Publication/

For manuscripts utilizing custom algorithms or software that are central to the research but not yet described in published literature, software must be made available to editors and reviewers. We strongly encourage code deposition in a community repository (e.g. GitHub). See the Nature Research guidelines for submitting code & software for further information.

Data

Policy information about availability of data

All manuscripts must include a data availability statement. This statement should provide the following information, where applicable:

- Accession codes, unique identifiers, or web links for publicly available datasets
- A list of figures that have associated raw data
- A description of any restrictions on data availability

Data relating to the findings of this study are available from the corresponding author upon request. Source Data have been deposited on GitHub alongside the analysis code: https://github.com/BeckerMatthias/MULTICOV-AB_Publication/

Field-specific reporting

Please select the one below that is the best fit for your research. If you are not sure, read the appropriate sections before making your selection.

- [x] Life sciences
- [ ] Behavioural & social sciences
- [ ] Ecological, evolutionary & environmental sciences

For a reference copy of the document with all sections, see nature.com/documents/nr-reporting-summary-flat.pdf

Life sciences study design

All studies must disclose on these points even when the disclosure is negative.

Sample size	Sample size were based on maximal available sample sets where detailed clinical and serological data were also available.
Data exclusions	No Data was excluded from analysis
Replication	For MultiCoV-Ab the %CVs of inter- and intra-assay precision were mostly below 10%, which justifies not repeating single sample measurements to save time and costs. Instead data reproducibility and assay consistency were assured by quality control measures as described in the manuscript. The commercial assays in this study passed the manufactueres quality control measures and were not repeated.
Randomization	No randomization steps were performed for experiments or data analysis. Samples were analyzed and grouped based on available metadata on SARS-CoV-2 infection, donor age, and time between infection and specimen collection. For the performed analyses no randomization was required.
Blinding	Blinding was not appropriate for this study of SARS-CoV-2 antibody responses in COVID-19 infected and uninfected individuals, with no associated therapeutic intervention. The primary objective of the study was diagnostic assay development.

Reporting for specific materials, systems and methods

We require information from authors about some types of materials, experimental systems and methods used in many studies. Here, indicate whether each material, system or method listed is relevant to your study. If you are not sure if a list item applies to your research, read the appropriate section before selecting a response.

Materials & experimental systems

n/a	Involved in the study	
[ ]	[x]	Antibodies
[ ]	[x]	Eukaryotic cell lines
[x]	[ ]	Palaeontology and archaeology
[x]	[ ]	Animals and other organisms
[ ]	[x]	Human research participants
[x]	[ ]	Clinical data
[x]	[ ]	Dual use research of concern

Methods

n/a	Involved in the study	
[x]	[ ]	ChIP-seq
[x]	[ ]	Flow cytometry
[x]	[ ]	MRI-based neuroimaging

Antibodies

Antibodies used	Antibodies bound to microspheres were detected with R-phycoerythrin labeled goat-anti-human IgG or IgA antibodies:<br>goat-anti-human-IgG-RPE (Dianova, Cat# 109-116-098, Lot#148837)<br>goat-anti-human-IgA-RPE (Dianova, Cat# 109-115-011, Lot#143454)<br>Penta-His Antibody (Qiagen, Cat# 34660)<br>donkey-anti-mouse antibody labeled with AlexaFluor647 (Invitrogen, Cat# A31571)
Validation	Both RPE-labelled detection antibodies used in the developed assay are widely used in assays based on xMAP Technology and are Immunoglobulin subtype specific as stated on the manufacturers website. We have further cross-validated the antibodies by measuring backgound signals when incubating with microshperes coated with human IgA or human IgG and did not find notable immunoglobulin-species cross-reactivity (data not shown).<br>The Penta-His and donkey-anti-mouse antibodies were used to confirm protein integrity by immunoblotting (data not shown). The antobodies are widely used for this application and have been validated in the literature and by the manufactureres, as stated on the manufacturers website.

Eukaryotic cell lines

Policy information about cell lines

Cell line source(s)	Expi293 were sourced from ThermoFisher.

Authentication	Cell lines were commercially purchased and no authentication was performed.
Mycoplasma contamination	The cell lines were not tested for mycoplasma contamination.
Commonly misidentified lines (See ICLAC register)	No commonly misidentified cell lines were used in this study.

Human research participants

Policy information about studies involving human research participants

Population characteristics	Donors with proven SARS-CoV-2 infection and SARS-CoV-2 unexposed individuals. A total of 1176 sera and plasma samples were used for the MultiCoV-Ab assay development. Only de-identified samples were used.<br>Samples were classified as SARS-CoV-2 infected, if a positive SARS-CoV-2 RT-PCR was reported and/ or if hospitalization/ quarantine for COVID-19 was indicated as part of the samples metadata.<br>Of the 1176 samples, 310 (26.4 %) samples were from COVID-19 patients or convalescents.<br>Cohort age was 5-88 years; age was not known for 161 samples.<br>Of the 1176 samples, 559 (47.5 %) were from male donors, 464 (39.5 %) were from female donors and for 153 (13.0 %) sex was unknown.<br>An detailed display of meta-data is shown in Supplementary Table 2.
Recruitment	All samples were pre-existing, no participants were recruited for this study.
Ethics oversight	Ethical approval was granted from the Ethics Committee of Hannover Medical School (#9122_BO_K2020).<br>SARS-CoV-2 infected samples used in this study were collected after ethical review (9001_BO_K, Hannover Medical School; 179/2020/BO2, University Hospital Tübingen; 85/20, Ärztekammer des Saarlandes).<br>Collection of non-SARS-CoV-2 infected control samples had been approved by several ethic committees: 3232-2016 (Ethics Committee of Hannover Medical School); 62/20 (Ethics Committees of the Medical Faculty of the Saarland University at the Saarland Ärztekammer); WUM 17.02.1997 (Joint ethics committee of the University of Münster and the Westphalian Chamber of Physicians)

Note that full information on the approval of the study protocol must also be provided in the manuscript.
